# Mind the translational gap: using iPS cell models to bridge from genetic discoveries to perturbed pathways and therapeutic targets

**DOI:** 10.1186/s13229-021-00417-x

**Published:** 2021-02-08

**Authors:** Greta Pintacuda, Jacqueline M. Martín, Kevin C. Eggan

**Affiliations:** 1 Department of Stem Cell and Regenerative Biology, Department of Molecular and Cellular Biology, Harvard Stem Cell Institute, Cambridge, MA 02138 USA; 2 Stanley Center for Psychiatric Research, Broad Institute of MIT and Harvard, Cambridge, MA 02142 USA

**Keywords:** iPSC (induced-pluripotent stem cells), NPC, Neurons, Differentiation, Neurodevelopment

## Abstract

Autism spectrum disorder (ASD) comprises a group of neurodevelopmental disorders characterized by impaired social interactions as well as the presentation of restrictive and repetitive behaviors. ASD is highly heritable but genetically heterogenous with both common and rare genetic variants collaborating to predispose individuals to the disorder. In this review, we synthesize recent efforts to develop human induced pluripotent stem cell (iPSC)-derived models of ASD-related phenotypes. We firstly address concerns regarding the relevance and validity of available neuronal iPSC-derived models. We then critically evaluate the robustness of various differentiation and cell culture protocols used for producing cell types of relevance to ASD. By exploring iPSC models of ASD reported thus far, we examine to what extent cellular and neuronal phenotypes with potential relevance to ASD can be linked to genetic variants found to underlie it. Lastly, we outline promising strategies by which iPSC technology can both enhance the power of genetic studies to identify ASD risk factors and nominate pathways that are disrupted across groups of ASD patients that might serve as common points for therapeutic intervention.

## Introduction

The parable of the blind men and the elephant has often been used as a metaphor for a reductionist cognitive process. Similar to the blind men trying to reconstruct the complexity of the elephant through fragmented experience, scientists aim at modeling complex human disease through functional studies of simpler, more approachable systems. In this perspective, availability of relevant experimental models is critical to further our understanding of any human disease, as well as to facilitate drug discovery. Advantages as well as potential limitations of employing human induced pluripotent stem cell (iPSC)-derived systems as an advanced technological tool to apply a reductionist approach to the study of autism spectrum disorder (ASD), have been extensively reviewed elsewhere [1–4]. Here, we focus on recent milestones in this space that are slowly enabling a more holistic outlook, and review efforts that apply iPSC-derived models to reconstruct, quantitate, and predict the complexity of the human brain. Specifically, we extensively discuss which cell types may be most vulnerable to the genetic variation underlying ASD and how their altered function might underlie behavioral changes in people with ASD. In this perspective, we critically evaluate neuronal iPSC-derived models used thus far to obtain such cell types in culture, and summarize reported cellular phenotypes that can represent partial readouts for ASD-like features. Then, we consider the few studies where iPSC technology has been coupled with human genetics to advance our understanding of the molecular makeup of neurodevelopmental complex genetic disease, including ASD. Finally, we contemplate ways that iPSC models can enhance the power of genetic studies to identify ASD risk factors and fuel discoveries of key pathways that are altered across many individuals with ASD and might serve as shared points for therapeutic intervention.

### Background: the advantages of modeling disease with induced Pluripotent Stem Cells (iPSCs)

Animal models, especially mice, have historically been a key tool in basic research and therapeutics. However, because of existing species-specific differences in pathways implicated in health and disease [5], human cell-based models have always been regarded as appealing complementary systems [6]. Human primary cells are however generally unavailable for study due to their limited availability from patients [7, 8].

In the attempt to overcome this impasse, a landmark in the field of cellular disease modelling was the employment of in vitro grown embryonic stem cells (ESC) derived from human blastocysts, that have the ability to indefinitely self-renew and can give rise to any type of somatic cells [9, 10]. However, despite providing many human cell types for research and therapeutics, their employment immediately raised societal concerns over their early embryonic origin [11], as well as practical limitations due to lack of information about the donors, and their familial and medical history (including their propensity to develop ASD) [12].

Takahashi and Yamanaka’s pioneering studies in the 2000s [13] led to rapid expansion of induced pluripotent stem cell (iPSC) technologies, and opened unprecedented opportunities for disease modeling. The last decade is testimony of how iPSC-based studies can enhance biomedical research and personalized regenerative medicine [12–14]: somatic cells from easily accessible tissues of patients can now be routinely reprogrammed into an embryonic stem cell-like state, and subsequently differentiated into cell types that are relevant for the same patient’s disease. iPSC technology also yields virtually unlimited amounts of human tissue carrying a genetic variant of interest, that becomes easily available for manipulation and therapeutic endeavors. Furthermore, genetic variants can be examined on a genetic background sensitive to the disorder and that may account for unpredicted secondary effects [15, 16].

The considerations above are all especially relevant in the context of neuropsychiatric and neurodevelopmental diseases—including ASD—which are for the most part quintessentially human and often polygenic [17, 18]. Primary cultures of patient-derived cells are largely unavailable, because brain biopsies for establishing an in vitro neuronal cell line, are considered unethical [19], while human *post mortem* samples, despite being desirable alternatives, typically do not represent the developmental stage when the disease is firstly manifested, and can be confounded by other factors, including treatment for the disease of study or for some of its symptoms [20]. Additionally, neither genetically engineered animal models, nor *post mortem* samples, have thus far had the capacity to predict patient-specific clinical outcomes to candidate ASD therapeutics [21].

**Fig. 1:**
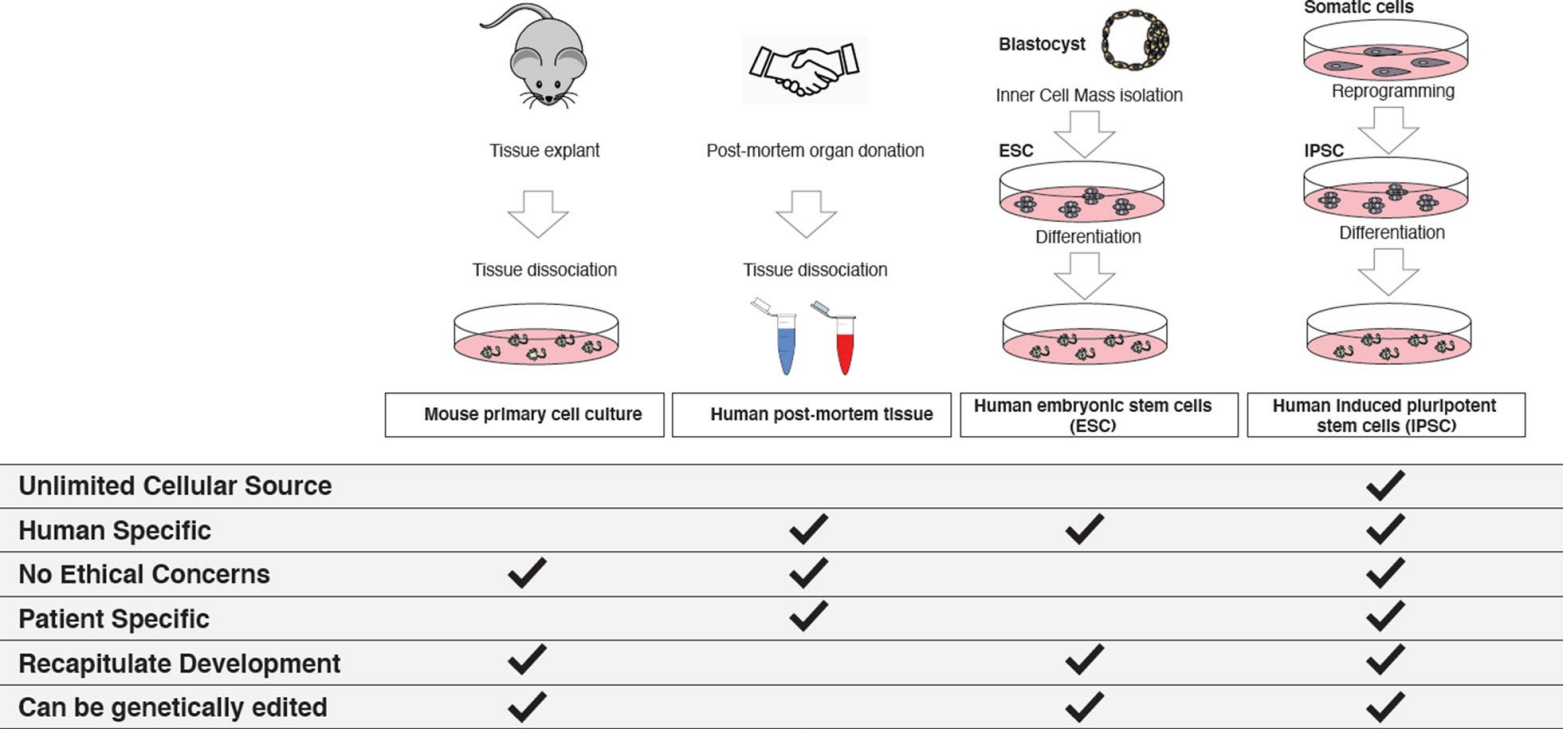
Overview of all available model systems currently employed to model disease. iPSC-based models represent a source of unlimited patient-specific material, able to recapitulate neuronal development without ethical concerns linked to use of embryonic material or patient biopsies

iPSCs meet all requirements to address these issues, as effectively unlimited quantities of patient-derived cells can be used to model cellular components of the human brain, to identify therapeutic targets, and to investigate said targets and design candidate therapies [2, 19–21] (Fig. 1). iPSCs are therefore an optimal resource to study various aspects of ASD in vitro, under the assumption that specific cell types are vulnerable to ASD, and that such cell types can be reliably derived from iPSCs using currently available protocols.

### ASD is a complex, polygenic, and heritable disorder

Under the broad diagnosis of ASD is a variety of neurodevelopmental disorders marked by impaired social skills and restrictive-repetitive behavior [3]. Individuals diagnosed with ASD exhibit a variety of phenotypes depending on a complex interplay between genetic and environmental factors and often manifest other comorbidities, both neurological and non-neurological.

The phenotypic complexity of ASD reflects its underlying genetic architecture, made of contributions from rare variants of large effect, either CNV (e.g., 16p11.2 or 22q11.2 duplication and deletion) or point mutations (e.g., CHD8, SCN2A), and common variants each conveying small effect but collectively shaping most of its risk [4, 22–26].

Recently, an unprecedented expansion of genome-wide association studies (GWAS) have led to the identification of common variants associated with ASD [22, 23, 27], while large-scale exome sequencing studies of ASD have now identified over 100 high-confidence autism risk genes [24, 25, 28]. However, how disruption of such genes results in altered neurodevelopment and neurophysiology in individuals with ASD, is still largely unclear.

Nevertheless, granular understanding of ASD genetic architecture has provided a tool in determining the dynamics of ASD onset during development at the cellular level, using analysis of concerted expression of ASD risk genes [29], and has been pivotal in defining the identity of cell types most relevant to ASD physiopathology. Identifying cell types that are vulnerable to ASD can subsequently guide efforts in perfecting protocols to derive such cell types from iPSC models [30], providing a promising avenue to translate genetic information into cell modeling.

### Cell types of both developing and adult brain are vulnerable to ASD and can be modelled in vitro

The phenotypic complexity of ASD suggests that there might be multiple cell types vulnerable to ASD both during development and adulthood (Table 1).

**Table 1:** ASD-vulnerable cell types (selected studies)

Cell type	Evidence	References
Excitatory/inhibitory neurons or NPCs	Enrichment of ASD risk genes in cell type-specific transcriptional modules	Parikshank et al. [29]
	Expression of modules of ASD risk genes in post-mortem cortical brain	Xu et al. [31]
	ASD-patient derived organoids produce an excess of GABAergic inhibitory neurons	Mariani et al. [145]
	Differentially expressed genes in transcriptomic data (ASD patient brain sample versus controls)	Gandal et al. [146]
	Expression of modules of ASD risk genes in single cell transcriptomic data of different human cell types	Wang et al. [147]
	Differentially expressed genes in single cell transcriptomic data (ASD patient cortex samples versus controls)	Valmesh et al. [36]
	Expression of modules of ASD risk genes in BrainSpa transcriptomic data and scRNA-seq data of human cortex	Satterstrom et al. [28]
Interneurons	GABAergic interneurons reduced in the autistic cerebral cortex	Hashemi et al. [148]
	Reduced number or activity in several mouse models	Filice et al. [149]
Sensory neurons	ASD-like behaviors in mice with conditional mutations of Mecp2, Gabrb3, or Shank3 in peripheral sensory neurons	Orefice et al. [49]
Oligodendrocytes	Dysregulation found in two ASD mouse models	Phan et al. [150]
Microglia	Deficient autophagy impairs synaptic pruning and induces behavioral defects in mouse models	Kim et al. [151]
	Differentially expressed genes in single cell transcriptomic data (ASD patient cortex samples versus controls)	Valmesh et al. [36]
Astrocytes	Interleukin-6 secretion from astrocytes in ASD individuals induces neural defects	Russo et al. [152]
Immune Cells	Neuroinflammation, autoantibodies, an elevated T cell response, an increase in NK cell and monocyte responses in mouse models	Mead et al. [153]

According to co-expression studies of ASD-relevant genes, a critical window for the onset of ASD, coincides with early fetal development, as specific molecular and cellular programs depending on coordinated expression of ASD risk genes, do not persist in the mature cells of the adult brain [29, 31, 32]. This argument nominates early time-points in neuronal maturation, such as Neural Progenitor Cells (NPCs), as attractive cell models. On the other hand, mature neurons have been strongly implicated in ASD physiopathology by a number of bulk gene expression studies showing changes in the neocortex of ASD patients, and indicating functional convergence of risk-gene expression in the adult brain [33].

Similarly, Satterstrom and colleagues, who applied co-expression network analysis to BrainSpan datasets, showed that ASD genes are indeed expressed at high levels not just in the developing brain, but also in the adult cortex [28]. The authors were able to pinpoint specific cell types, based on expression modules of 4,261 cells from the prenatal human forebrain [34]. In accordance with previous evidence that identified excitatory glutamatergic neurons [29, 31, 32], they found neuronal cell types as being prevalently recapitulating the transcriptional signature of ASD, with most genes being expressed in excitatory and, to a lesser-extent, inhibitory lineage cells [28]. Other neuronal types found to be enriched for ASD signal were striatal interneurons. Interestingly, Cogill et al. demonstrated that also lncRNAs may play a role in ASD due to their convergence on shared pathways with ASD-associated coding-genes. It will be important to follow up on this finding and include lncRNAs in future re-analyses based on co-expression modules [35]. Despite the general lack of statistical power in molecular studies involving scarcely available post-mortem tissue, Velmeshev and colleagues recently conducted a single-nucleus RNA sequencing study on cortical tissue from patients, and found that expression of synaptic and neurodevelopmental genes is especially affected in cortical neurons [28].

In conclusion, although some non-neuronal cell types were found to be vulnerable to ASD (including microglia and OPCs), most of the transcriptional modules seem to converge on neuronal lineages, and notably on maturating neurons and excitatory neurons of the adult neocortex [36].

It should also be noted that abnormal pain sensitivity is commonly reported in ASD patients [37] and developmental disorders linked to monogenic forms of ASD are also associated with defects in somato-sensation [38]. Many studies conducted in rodent models of ASD with highly penetrant monogenic mutations, indicate that abnormalities in sensory reactivity correlate with ASD-related phenotypes, in line with the hypothesis that impaired sensory perception may impact brain development and function, and results in disparate symptoms associated with ASD [39]. This hypothesis provides a potential mechanistic link between otherwise heterogeneous ASD-related phenotypes, and implicates peripheral sensory neurons, in addition to the other neuronal types discussed above, in ASD etiology [40].

### State-of-the art iPSC-derived differentiation protocols that model cell types vulnerable to ASD

iPSC-based differentiation protocols offer a valuable resource to generate cell types relevant to virtually any disease of interest, with the caveat that such cell types can be reliably derived from iPSCs using currently available protocols.

A selected overview of the otherwise large number of published protocols for neural differentiation of cell types relevant to ASD, is reported in Table 2. They are all based on the premise that it is possible to mimic embryonic differentiation in a dish, with the distinction that some recapitulate intermediate NPC states, while others achieve direct differentiation to the terminal neural cell type of choice. Either way, many protocols start with dual SMAD inhibition [41]. Subsequent differences in concentration of patterning factors or in the timing of their addition can yield a variety of neural cell types and impact largely the homogeneity and nature of neurons. In order to overcome culturing heterogeneity, many protocols are now based on inducible transcription factor expression, via virus transduction or integration into a safe harbor locus [42–46]. These protocols generally produce highly differentiated and homogeneous cells in a shorter time frame and on a larger scale when compared to others. However, it is still essential to share detailed experimental guidelines to guarantee reproducibility of each new protocol.

**Table 2:** Protocols for fast generation of specialized neurons from iPSC cells (selected studies)

Terminal cell type	Protocol method	References
Glutamatergic excitatory neurons and neural progenitors	NGN2 expression	Zhang et al. [154]
	NGN2 expression and WNT/dual-SMAD inhibition	Nehme et al. [47]
GABAergic inhibitory neurons	Transient expression of TFs (Ascl1 and Dlx2)	Yang et al. [155]
Dopaminergic neurons	Transient expression of TFs (rLmx1a, rNurr1 or rPitx3)	Mahajani et al. [156]
Sensory peripheral neurons	Small molecule-mediated direct differentiation, followed by human epidermal keratinocytes-conditioned medium	Guimareãs et al. [157]
Interneurons	Small molecule-mediated direct differentiation	Maroof et al. [158]

High degree of reproducibility was reported by Nehme and colleagues, who combined small-molecule with transcriptional patterning, to generate cortical excitatory glutamatergic neurons [47]. After three weeks of maturation, transcriptomic analysis confirmed homogenous maturation of cultured iPSCs into upper layer cortical projection neurons. Microelectrode array and patch clamp electrophysiology also showed AMPA and NMDA-mediated synaptic transmission, which are hallmarks of postnatal cortical neurons. Of particular interest for NPC in vitro modelling, Wells and colleagues adapted the protocol developed by Nehme [47], allowing rapid (48 h) generation and maintenance of human stem cell-derived progenitor cells (SNaPs) [48].

Finally, the association between ASD and altered somato-sensation, suggests that generating iPSC models of sensory neurons from ASD patients will be a valuable system for testing the ability to reverse some ASD-related cellular phenotypes, as previously done in mouse models [49], and spinal cord [50]. Although few protocols have been previously reported (Table 2), none has been utilized in ASD research thus far.

### Complex cell culturing systems to study ASD

**Fig. 2:**
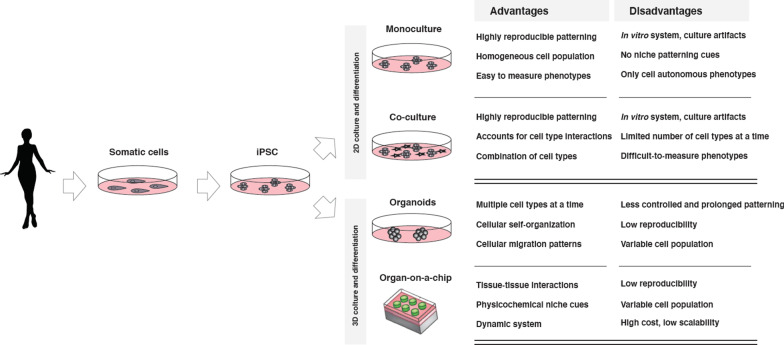
Summary of various iPSC-based culturing systems. Bi-dimensional cultures can be adapted for co-culturing of more than one cell type at a time. Three-dimensional cultures can be supported by microfluidic devices

The translational potential of iPSC-derived models can be further enhanced by complementing cell culture with the inclusion of additional components of the in vivo niche of the cell type of interest, or mimicking cell–cell and cell–matrix interactions that occur within organs and tissues [51] (Fig. 2). Complex culturing systems, including co-cultures and three-dimensional (3D) cultures, may also account for non-cell-autonomous effects on differentiation, and help modulate neuronal activity and drug response, while also promoting neuronal maturity [52].

One of the most ASD-relevant examples of co-cultures consists of iPSC-derived neurons and glial cells, an abundant cell population in the human brain that have critical supporting roles for neurons in both health and disease [53, 54]. Co-culturing neurons with microglia has been key in studying chronic inflammation correlated with ASD and neurodegeneration [55]. Other examples of cell types that have been co-cultured with neurons in ASD modeling are oligodendrocytes (impacting neuronal myelination [56]) and astrocytes (impacting viability, synaptic function, and neurite outgrowth [57]).

3D culturing conditions are also an important development for improving physiologically-relevant in vitro disease models. For instance, it is now possible to model specific regions of the brain, allowing a more holistic comparison between ASD-derived and control cultures [58]. Although 3D cultures can be achieved through microfluidics and bioprinting, one of the most promising technologies is that of iPSC-derived brain organoids. Brain organoids consist of multicellular aggregates that differentiate and self-organize, mimicking its in vivo development [59], and offer new models for assessing the pathogenesis of ASD, especially in the context of monogenic syndromes [60–63]. Additionally, implementations of organoid protocols allow production of specific regions of the brain, including hippocampus and cerebellum, as well as cortical folding, enabling a holistic study of the human brain in development and disease [58]. Attempts have also been made to use this technology to recreate early stages of corticogenesis, particularly relevant to the study of prenatal brain organization and function [64].

Co-culture and three-dimensional (3D) cultures are therefore a promising development for iPSC modeling of complex tissues (reviewed in [65, 66]), although currently still challenging to establish and reproduce, mostly due to batch-to-batch or organoid-to-organoid heterogeneity and long differentiation periods (60–120 days to reach differentiation levels similar to mid-gestation, compared to 14–30 days of NGN2-based patterning protocols).

### iPSC-based models manifest ASD-related, measurable phenotypes

NPCs and neurons derived from syndromic and idiopathic individuals diagnosed with ASD, display a wide range of phenotypes [30, 67–70]. The phenotypic diversity observed in many individuals with ASD is representative of the underlying heterogeneity of their genetic background and is also reflected on the diversity of reported cellular phenotypes observed across iPSC models. Comprehensive catalogues of ASD-relevant cellular phenotypes as well as detailed description of current Biobanks of deposited patient-derived cell material, have been compiled in a number of excellent reviews [71, 72]. However, it is often challenging to compare qualitative observations, and the field would certainly benefit form adapting standardized quantitative measures to evaluate the impact of genetic background on cellular phenotypes.

**Fig. 3:**
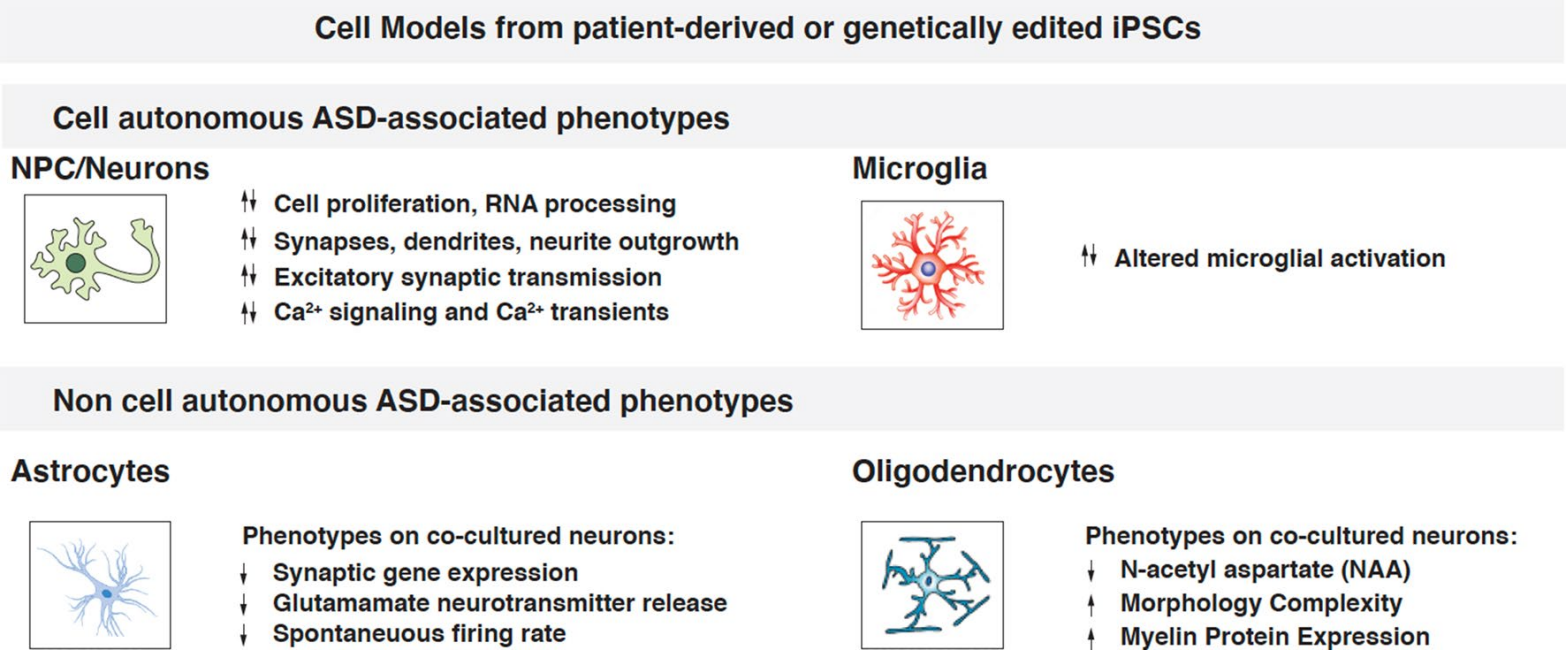
Overview of measurable phenotypes observed in cell types either derived from ASD patients or obtained via gene-editing. Neuronal phenotypes can be cell autonomous or mediated by interaction with co-cultured non-neuronal cells

**Fig. 4:**
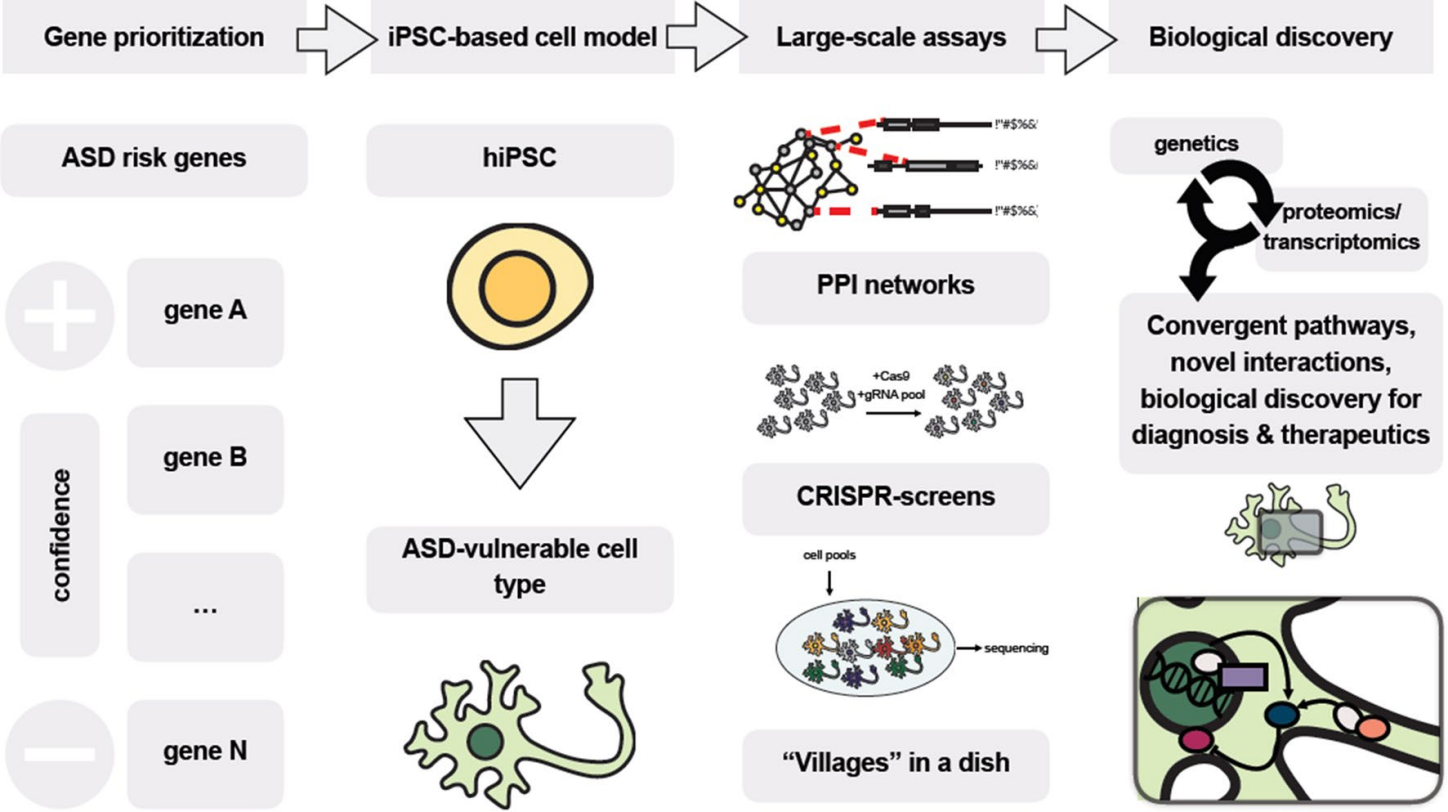
iPSC-based models as fundamental tools to bridge human genetics and functional studies in ASD, through the employment of large-scale assays, including PPI networks, CRISPR-screens and “villages”

Modelling ASD with iPSC technology and classifying each model based on a well-defined subset of quantitative qualifiers or “phenotypic classes”, has proven crucial in revealing novel cellular and molecular mechanisms underlying it. Thus far, the most robust quantitative measures utilized to stratify cellular phenotypes have focused on cell proliferation and brain growth; RNA-processing; synapse density and dendritic arborization; electrophysiology; and calcium signaling (Fig. 3). Here we provide a summary of the most reproducible, robust, and representative cellular phenotypes according to these metrics (Table 3), and focus on few representative examples to discuss how they relate to ASD symptoms, and whether they can be used for translational endeavors (Fig. 4).

**Table 3:** Summary of published studies using iPSC-based models to study ASD (alphabetical order)

Mutation	Donors (cases/controls)	Isogenic (yes/no)	Class of observed “phenotypic classes” (G, R, E, S, C)	References
15q13.3	6/3	No	E, C	Gillentine et al. [159]
16p11.2	6/3	No	G, E, S	Deshpande et al. [79]
22q11.2	8/7	No	G	Lin et al. [160]
CACNA1C	2/2	No	E	Krey et al. [98]
CACNA1C	2/3	No	C	Pasca et al. [161]
CDK5RAP2	4/4	Yes	G	Lancaster [60]
CHD8	2/4	Yes	G	Wang et al. [162]
DYRK1A	105 patients	No	G	Courcet et al. [77]
FMR1	3/1	No	E	Doers et al. [97]
FMR1	1/1	Yes	R	Lu et al. [92]
FMR1	2/2	Yes	R	Sunamura et al. [90]
MECP2	2/1	No	E	Nageshappa [95]
Multiple genes	15/11 (53 lines)	Yes	E	Deneault et al. [104]
Multiple genes	1/1 per gene	Yes	C	Deneault et al. [101]
NLGN4	2/1	Yes	E, S	Marro et al. [163]
NRXN1	3/5	No	E, C	Avazzadeh et al. [106]
NRXN1	4/4	Yes	E, S	Flaherty et al. [164]
NRXN1	1/4	No	G, E, C	Lam et al. [165]
NRXN1	2/1	Yes	E	Pak et al. [102]
PTCHD1-AS	2/2	Yes	G, E, S	Ross et al. [103]
SHANK2	2/4	Yes	G, E, S	Zaslavsky et al. [100]
SHANK3	4/3	No	S	Gouder et al. [166]
SHANK3	1/1	Yes	E, S	Huang et al. [167]
SHANK3	2/3	Yes	S	Kathuria et al. [168]
SHANK3	1/1	Yes	E	Yi et al. [96]
UBE3A	3/4	Yes	E	Fink et al. [169]
UBE3A	1/1	Yes	E	Sun et al. [170]
Idiopathic	7/6	No	G	Courchesne et al. [73]
Idiopathic	5/5	No	E, C	DeRosa et al. [107]
Idiopathic	6/6	No	R	Griesi-Oliveira et al. [83]
Idiopathic	1 family	No	G	Lewis et al. [171]
Idiopathic	3/3	No	E, S	Liu et al. [172]
Idiopathic	8/5	No	G, E, S	Marchetto et al. [69]
Idiopathic	4/8	No	G, E, S	Mariani et al. [145]
Idiopathic	3/3	No	G, S	Moore et al. [173]
Idiopathic	3/3	No	G, E, S	Russo et al. [152]
Idiopathic	8/5	No	S	Schafer et al. [174]
Idiopathic	3/3	No	G	Wang et al. [80]
Idiopathic/PTEN	3/15	No	G	Butler et al. [76]

To compare observed phenotypes, they were categorized based on five “phenotypic classes”: G = cell proliferation and brain growth; R = RNA-processing; S = synapse density and dendritic arborization; E = electrophysiology; C = calcium signaling

#### Cell proliferation

Several clinical studies of ASD have reported accelerated brain growth in the first three years of life of patients [73]. This translates in macrocephaly, that is in fact a characteristic phenotype of some genetic subtypes of ASD. Conversely, microcephaly, the inverse phenotype, is also associated with autism. For example, deletions and duplications at two loci, 1q21.1 and 16p11.2, have opposing brain growth phenotypes [74], as well as individual gene mutations: CHD8′s [75] and PTEN’s [76] genetic variants are associated with macrocephaly, while DYRK1A’s [77] and CDKL5′s [78] with microcephaly. iPSC models of rare microcephalic syndromes recapitulate loss of NPCs and premature neural differentiation [60], while iPSC-derived NPCs from subjects with ASD and macrocephaly, display rapid proliferation [69]. Similarly, cellular models of deletions and duplications of 16p11.2, recapitulate opposite effects on cell proliferation while not significantly affecting synaptic density [79]. Recently, Wang and colleagues suggested that accelerated proliferation of iPSC-derived NPCs from ASD individuals with macrocephaly can be linked to altered DNA replication and increased DNA damage [80]. Taken together, these studies suggest that iPSC models can be used to study the effects of different mutations on cellular phenotypes, while standardized assays for cell proliferation and growth on patient-derived NPCs could represent proxies for underlying genetic syndromes.

#### RNA-processing

Since the early days of gene expression analysis, differences between ASD patients and controls measured with microarrays, identified gene splicing as one of the biological processes defective in several forms of autism [81]. Since then, defects in RNA splicing and processing have been consistently reported in many studies [82, 83], and even proposed as potential ASD biomarkers [84, 85]. Additionally, syndromic and idiopathic forms of ASD have been linked to dysfunction of RNA metabolism [86, 87], with a number of ASD-risk genes found to either encode for or regulate RNA-binding proteins, long non-coding RNAs (lncRNAs), and transcriptional regulatory elements [28, 88]. Human regulatory elements and non-coding RNAs are often poorly conserved in mice or rats, and there are interspecies differences across vertebrates for mechanisms controlling the expression of conserved protein-coding genes [89]. These arguments indicate human iPSC-derived cell models as the most suitable system to study ASD-related phenotypes linked to gene expression regulation and RNA processing. In this respect, most recent work has focused on the central role of the FMR1 mutation in FXS, the most common inherited form of intellectual disability frequently associated with autism. It was observed that NPCs derived from FMR1-knockout iPSCs display altered expression of neural differentiation markers [90], and that FMR1 deficiency in iPSC derived from FXS patients as well as in embryonic stem cells derived from FXS blastocysts has significant impact on gene expression patterns during neuronal differentiation [91, 92].

A more comprehensive characterization of such targets in FXS patients as well as on other individuals with idiopathic and genetically-profiled ASD will be key in discovering potential candidate genes for therapeutic and diagnostic purposes. Furthermore, a human-specific roadmap of pathways that are co-regulated by shared RNA-processing machinery in ASD cell models, could provide an additional tool for patient stratification and offer easily detectable biomarkers [93].

#### Synapse density and dendritic arborization

Seminal studies on the biology of MECP2 were performed on iPSC-derived neural cells obtained from Rett syndrome patients carrying loss-of-function MECP2 mutations. Cortical neurons derived from patients show reduced arborization and less glutamatergic synaptic puncta, resulting in impaired neural networks [68]. Conversely, cell models of MECP2 gain-of-function (MECP2 duplication syndrome), show increased synapses and dendrites [94, 95]. Reduced dendritic arborization, excitatory synapses, and neurite outgrowth, was also observed in iPSC models of other syndromes with ASD-like symptoms, including models of SHANK3 [70, 96], FMR1 [97], and CACNA1C [98]. Increased dendrite length and synaptogenesis has instead been reported in neuron models of Williams syndrome [99] and SHANK2 [100]. These observations further highlight how different mutations even within the same gene can have measurable phenotypic effects on a cellular level.

#### Electrophysiology

Deneault and colleagues made use of CRISPR/Cas9 technology to generate iPSC lines carrying mutations in ASD-associated genes, including ATRX, AFF2, KCNQ2, SCN2A [101]. Subsequent patch-clamp recordings on each edited cell line showed reduced excitatory post-synaptic potentials (EPSPs) when compared to isogenic controls. Similarly, iPSC-derived neurons from ASD patients, revealed significant deficits in excitatory synaptic transmission, that was recovered by forced expression of SHANK3 as well as by pharmacological treatment with IGF-1. This observation indicates that the synaptic defects observed in SHANK3 animal models, which have been functionally tied to failure in proper organization of HCN-channels [96], can be potentially treated both pharmacologically and genetically [70]. Impaired synaptic function was also observed in ESC-derived neurons carrying a heterozygous mutation of the gene NRXN1. However, in this case, the underlying phenotype was explained by defects in neurotransmitter release rather than neuronal differentiation or synapse formation [102]. Similarly, several deletions within the PTCHD1 gene result in diminished excitatory postsynaptic current frequency [103]. On the opposite side of the spectrum, neuronal models of SHANK2 [100], CNTNAP5, and EHMT1 [104] display hyper-connectivity. Generalizing all above observations, electrophysiological activity of neurons represents a readout that could potentially identify genes that cause synaptic phenotypes and offers opportunities to generate platforms to test the effects of genetic manipulation and pharmacological intervention [105].

#### Calcium signaling

Several studies showed abnormalities in calcium signaling and calcium transients in ASD patients, nominating calcium imaging as a powerful readout for ASD-relevant cellular phenotypes, albeit often challenging to optimize for in vivo studies. iPSC-derived neuronal cultures are homogenous and monolayered, and therefore optimal systems for calcium indicator visualization. Avazzadeh and colleagues, utilized iPSC lines derived from 5 healthy controls and 3 ASD individuals carrying heterozygous mutations within the NRXN1 gene, one of the most prevalent genes associated with monogenic ASD as well as other neuropsychiatric and neurodevelopmental diseases. Interestingly, all iPSC lines derived from patients carrying a heterozygous mutation for the α isoform of the NRXN1 gene (NRXN1α^+/−^), presented with an upregulation of voltage-gated calcium channels as well as an increased Ca^2+^ transients [106]. Furthermore, DeRosa and colleagues reported a decrease in spontaneous Ca^2+^ transient events at specific time points during neuronal maturation of patient-derived iPSCs, using multi-electrode array recordings [107]. These results suggest that calcium imaging-based assays can be successfully utilized as readouts for ASD-related cellular phenotypes.

### iPSC-based models as fundamental tools to bridge human genetics and functional studies in ASD

Experimental data of protein–protein interaction (PPI) networks have been mathematically modelled using topological structures [108]. In the context of several human genetic diseases, PPI networks helped clustering modules of proteins encoded by risk genes based on their interactions, thus providing a tool to identify functional convergence [105–111]. In the ASD-modeling space, Neale and colleagues showed a highly significant enrichment of ASD de novo variants within the PPI network connecting genes mutated in familial ASD [112]. It should be noted that for this seminal study, the authors made use of a database collecting experimental PPI datasets independently of their biological source [113]. Lage and colleagues, however, showed that a much more relevant enrichment for genetic signal was observed when only PPIs obtained from cell types and tissues relevant to the disease, were considered [114]. This result highlights the importance of promoting a global effort to generate and share high-quality PPI data in relevant cell types, in order to potentially identify functional hubs where ASD genetic signal might converge [115]. iPSC-derived cell models are currently a valuable source of scalable cellular material compatible with proteomic analysis, and provide a tool to translate genetics into biological discovery [115].

ASD genetics can also be coupled to iPSC-modelling through Massive Parallel Reporter Assays (MPRAs) and CRISPR-screens coupled to large-scale sequencing (Table 4). In the CRISPR-Cas9 system, a guide RNA (gRNA), in complex with the Cas9 protein, targets genomic sequences homologous to the gRNA and modifies the gRNA-targeted DNA sequence, enabling “surgical” genome-editing [116]. Notably, Cas9 also allows for multiplexed targeting via co-delivery of pooled libraries of gRNAs [117], and can be modified in its catalytic activity to modulate gene expression rather than generating a genetic scar [118]. The challenges and strength of CRISPR-based functional genomics in iPSC-derived disease models have been discussed in excellent reviews [119, 120], and include the ability of designing ad hoc gRNAs targeting extensive sets of genetic variants, and screen for loss-of-function, gain-of-function and haploinsufficiency. Specifically, in the ASD modeling space, one can think of applying CRISPR screens to iPSC-derived cell cultures, simultaneously perturbing large sets of ASD risk genes and utilizing quantitative standardized assays (including the ones mentioned in the previous section) to assess associated cellular phenotypes [119]. This seems especially relevant in light of a recent study by Tian and colleagues, showing that CRISPR interference platforms can in fact be used for genetic screens in human iPSC-derived neurons [121].

**Table 4:** Representative studies of large-scale forward-genetic methods applied to cellular system, that can be (or have been already) adapted to couple iPSC-derived neuronal cell models to ASD genetics

Platform	Technology	Short description	References
MPRAs	Saturation mutagenesis with MPRAs	Mutagenesis on disease-associated gene promoters and enhancers	Kircher et al. [175]
	Targeted variants mutagenesis with MPRAs	Functional dissection of common genetic Variation	Ulirsch et al. [176]
CRISPR screens	Perturb-seq	CRISPR screen combined with single cell RNA-seq (scRNA-seq)	Dixit et al. [122]
	CRISPRa/i screens	CRISPR screens modulating gene expression	Tian et al. [177]
	CREST-seq	Cis-regulatory elements scan by tiling-deletions	Diao et al. [178]
	MOSAIC-seq	Genome-wide CRISP/i screens targeting enhancers	Xie et al. [179]
	CRISPR-flowFISH	RNA-FISH coupled to genome-wide CRISP/i screens targeting enhancers	Fulco et al. [180]
CRISPR targeting	CRISPRa/i gene targeting	CRISPRa/i-mediated modulation of selected regulatory regions	Gasperini et al. [181]
	CRISPR-mediated allelic replacement	CRISPR-mediated nonhomologous end joining (NHEJ) or homology-directed repair (HDR)	Ran et al. [117]

Furthermore, whole transcriptome sequencing can be coupled to a CRISPR-screen (Perturb-seq) to measure the overall changes in molecular pathways prompted by each individual mutation at the population (bulk RNA-seq) and single cell (scRNA-seq) level [122]. It should be noted that a similar approach to study ASD-related genes in iPSC models, has been proposed as part of the Psychiatric Cell Map Initiative [119, 123], and has been already successfully employed to map genetic networks in human cells [124], and in animal models. Notably, Jin and colleagues used in vivo Perturb-seq to introduce frame shift mutations in 35 genes strongly associated with ASD in humans, and studied their effect on mouse postnatal brain [125]. Given the recent advances in adapting CRISPR-screen to human neurons [126], it is easy to imagine how a similar experimental design could be soon translated to iPSC-derived human models.

Additionally, a number of studies have recently focused on employing genetic heterogeneity within populations, to exploit the multiplexing potential of single cell RNA-seq [127]. Specifically, genetic information offers a natural identifier or barcode to demultiplex pooled samples, allowing complex combinatorial experimental designs of single cell RNA-seq experiments. This approach has been successfully combined to CRISPR-screens, and iPSC-derived neurons represent an ideal cell model to analyze effects of ASD-risk genes on cellular phenotypes [128]. Another interesting application of recent advances in pooled RNA-seq technology is represented by systematic sequencing of villages of neurons obtained from patient-derived iPSCs [129, 130]. For instance, Cederquist and colleagues, pooled in a single dish 30 isogenic iPSC lines harboring de novo ASD mutations to disentangle ASD genetic heterogeneity [131]. The same approach can be used to explore polygenic risk or to assess the impact of genetic background on ASD-relevant phenotypes and eQTLs (expression quantitative trait loci). Lines carrying monogenic ASD mutations of patients that have been extensively genotyped certainly represents an attractive proof-of-principle for further developments of this approach.

### Future perspectives: iPSC models to drive therapeutic intervention

In this review, we summarized how iPSCs have been utilized to model certain aspects of ASD, and to quantitatively assess ASD-associated phenotypes. Looking into the future, it will be important to enhance the translational potential of current technologies. Given the heritability of ASD, gene therapy offers a complementary alternative to small molecule-based approaches, especially in the monogenic syndrome space. This approach has been already largely explored for treatment of several diseases and notably for some neurological monogenic disorders [132, 133]. Genetic correction could be tested on cell systems to optimize both optimal carriers and efficiency in rescuing specific phenotypes. AAVs have emerged as the principal delivery candidates, and have proven effective in mice carrying a null allele for *Mecp2* [134, 135]. However cell toxicity, optimal time-window for transduction, and off-target effects have not yet been determined, and need further investigation [136]. iPSC models represent an immediate venue for these types of evaluations. An appealing alternative to genetic correction is modulation of gene expression by knockdown of mRNA transcripts through antisense oligonucleotides (ASOs) or short interfering RNAs (siRNAs). Both technologies are based on Watson–Crick base pairing to particular mRNA transcripts aimed at preventing their translation (detailed mechanisms of action, are reviewed elsewhere [137]). ASOs targeting *Ube3a-ATS* have been used to correct cognitive deficits in a mouse engineered to model features of Angelman syndrome [138]. In the same vein, ASOs have been used to normalize MeCP2 levels and rescue the neurological defects observed in mice carrying an extra copy of *MECP2* [139]. This avenue appears to be broadly attractive for treatment of many syndromes caused by haploinsufficiency, where effective therapeutics should aim at restoring normal range of gene expression rather than editing the genome.

Although humanized mouse models, circumventing the issue of potential interspecies differences across vertebrates, may be utilized for testing these studies, patient-derived iPSC models represent a more expedited and scalable tool to test transability of these results in humans and vulnerable cell types, as in the case of other neurological and neurodegenerative conditions, including ALS/FTD and AD [140, 141].

## Conclusions

ASD comprises a group of highly inheritable neurodevelopmental disorders characterized by impaired social interactions as well as the presentation of restrictive and repetitive behaviors [3]. The phenotypic complexity of ASD reflects its underlying genetic architecture, made of contributions from highly penetrant rare variants, and common variants each conveying small effects but collectively shaping most of its risk [4, 22–26].

In this review, we discussed how iPSC technology has become central to modelling various aspects of complex human disease, and can potentially allow researchers to advance our understanding of the pathophysiology of ASD, and to test personalized drug candidates. However, since the goal of iPSC-based ASD models is to reproduce and somehow functionally break down the complexity of the human brain, they need to be highly elaborate, yet reproducible. As a consequence, there are many challenges to face, including improving reliability and robustness of iPSC culturing and differentiation protocols. In this perspective, a good balance should be found in the current attempts on advancing the complexity of cellular models [142], and the vast batch effects, that might account for the most part of measured phenotypes [143].

Development of more robust protocols, employment of isogenic lines, and use of gene editing to compare variants within the same genetic background, will certainly contribute to overcome most technical roadblocks encountered in the past [118–120]. The rapid development of protocols to derive three-dimensional cultures and organoids, as well as to efficiently maintain co-cultures of mixed cell-types, coupled with technological advances in engineering culturing devices, all seem to be promising paths towards obtaining more complex and accurate disease models [60–62, 144]. Additionally, iPSC biobanks, providing access to a plethora of established, well-characterized and well-annotated iPSC, have significantly improved our understanding of the biological basis of natural genetic variation [117–119].

Generating faithful models is of utmost importance, but efforts in obtaining more reliable cell models, must be matched with advances in standardizing measurable cellular phenotypes related to certain aspects of ASD. In fact, the community would immensely benefit from a standardized assessment of in vitro phenotypes that reflect disease-relate mechanisms rather than generic experimental and/or genetic variance. Individual studies, mostly performed on iPSC-derived models of monogenic syndromes, have focused on assaying cell-proliferation, altered RNA-processing, electrophysiological properties, synaptic structure and calcium signaling [68, 69, 104, 106]. Assessing these phenotypes in larger-scale studies, comparing vast numbers of iPSC-derived cell lines, and linking them to dysfunction of discrete molecular pathways, is more than ever necessary. In parallel, continuous efforts in clinical sequencing of stratified patients and broadening biobank databases, will be key in advancing our understanding of complex genotype–phenotype correlations at the individual and cellular levels [48, 129].

In conclusion, recent advances in the field of human genetics, with tens of genes being identified as concentrating ASD risk [27], and hundreds of rare-variants with different degrees of penetrance [28], emphasized the need for a better understanding of the complexity of ASD. iPSC-based cell systems, while offering an unprecedented opportunity for modeling measurable ASD-related phenotypes, also provide a unique platform to rapidly validate and enhance genetic findings by nominating pathways that are disrupted across groups of ASD patients [48, 129]. These might represent as hot-spots for ASD vulnerability and desirable targets for therapeutic intervention.

## Data Availability

Not applicable.

## Abbreviations

AD: Alzheimer disease
ALS: Atrophy lateral sclerosis
ASD: Autism spectrum disorder
CNV: Copy number variant
CRISPR: Clusters of regularly interspaced short palindromic repeats
eQTL: Expression quantitative trait locus
ESC: Embryonic stem cell
FXS: Fragile X syndrome
FTD: Frontotemporal dementia
GWAS: Genome-Wide Association Study
iPSC: Induced pluripotent stem cell
MPRA: Massive parallel reporter assay
NPC: Neural progenitor cell
PPI: Protein–protein interaction
OPC: Oligodendrocyte progenitor cell
scRNA-seq: Single-cell RNA-sequencing
